# Variation in spatial and temporal incidence of the crustacean pathogen *Hematodinium perezi* in environmental samples from Atlantic Coastal Bays

**DOI:** 10.1186/2046-9063-9-11

**Published:** 2013-05-04

**Authors:** Ammar W Hanif, Whitney D Dyson, Holly A Bowers, Joseph S Pitula, Gretchen A Messick, Rosemary Jagus, Eric J Schott

**Affiliations:** 1 Institute of Marine and Environmental Technology, University of Maryland Center for Environmental Science, Baltimore, MD, 21202, USA; 2 Department of Natural Sciences, University of Maryland Eastern Shore, Princess Anne, MD, 21853, USA; 3 Monterey Bay Aquarium Research Institute, Moss Landing, CA, 95039, USA; 4 Cooperative Oxford Laboratory, Center for Coastal Environmental Health & Biomolecular Research, USDOC/NOAA/NOS/NCCOS, Oxford, MD, 21654, USA

**Keywords:** Blue crab, *Hematodinium*, Parasite, Disease reservoir, Fishery

## Abstract

**Background:**

*Hematodinium perezi,* a parasitic dinoflagellate, infects and kills blue crabs, *Callinectes sapidus,* along the Atlantic and Gulf coasts of the United States. The parasite proliferates within host hemolymph and tissues, and also produces free-swimming biflagellated dinospores that emerge from infected crabs. Infections in *C. sapidus* recur annually, and it is not known if biotic or environmental reservoirs contribute to reinfection and outbreaks. To address this data gap, a quantitative PCR assay based on the internal transcribed spacer 2 (ITS2) region of *H. perezi* rRNA genes was developed to asses the temporal and spatial incidence of the parasite in Delaware and Maryland coastal bays.

**Results:**

A previously-used PCR assay for *H. perezi*, based on the small subunit rRNA gene sequence, was found to lack adequate species specificity to discriminate non-*Hematodinium* sp. dinoflagellate species in environmental samples. A new ITS2-targeted assay was developed and validated to detect *H. perezi* DNA in sediment and water samples using *E. coli* carrying the *H. perezi* rDNA genes. Application of the method to environmental samples identified potential hotspots in sediment in Indian River Inlet, DE and Chincoteague Bay, MD and VA. *H. perezi* DNA was not detected in co-occurring shrimp or snails, even during an outbreak of the parasite in *C. sapidus*.

**Conclusions:**

*H. perezi* is present in water and sediment samples in Maryland and Delaware coastal bays from April through November with a wide spatial and temporal variability in incidence. Sampling sites with high levels of *H. perezi* DNA in both bays share characteristics of silty, organic sediments and low tidal currents. The environmental detection of *H. perezi* in spring, ahead of peak prevalence in crabs, points to gaps in our understanding of the parasite’s life history prior to infection in crabs as well as the mode of environmental transmission. To better understand the *H. perezi* life cycle will require further monitoring of the parasite in habitats as well as hosts. Improved understanding of potential environmental transmission to crabs will facilitate the development of disease forecasting.

## Background

The blue crab, *Callinectes sapidus*, is an ecologically and economically significant crustacean with a geographic distribution from maritime Canada to Uruguay. It is a key epibenthic link in the near shore marine environment where it feeds on a diverse range of invertebrates, and is a component of the diet of many coastal fish species. In the Atlantic and Gulf coast of the United States the blue crab supports a commercial fishery with an estimated worth of $165 million/year [1] and a substantial recreational fishery of undetermined magnitude that extends as far north as Massachusetts. There was a time when the blue crab was so abundant the harvests were described as “inexhaustible” [2]. However, the blue crab population in the Chesapeake Bay has been at historic lows for most of the past decade, most likely due to levels of exploitation [3,4]. Other states such as North Carolina, South Carolina and Georgia have also experienced declines in their blue crab population and harvests [5]. In the Chesapeake Bay, the persistently low blue crab broodstock stimulated the formation of a research consortium to study hatchery-based broodstock enhancement and motivated Maryland and Virginia to implement unprecedented harvest restrictions [6,7]. Though harvest restrictions have helped alleviate the strain on the blue crab population, it is typical to have year-to-year fluctuations in abundance that cannot be explained by fishing pressure alone. It is now understood that estimates for natural mortality of blue crab are over-simplified and even though many blue crab diseases have been described, their influence on mortality and population structure is poorly understood [8-11].

In mid-Atlantic coastal bays of North America, blue crab populations suffer episodic mortalities associated with infections by a parasitic dinoflagellate in the genus *Hematodinium*[12,13]. Currently, there are two formally described species in the genus: *H. perezi* and *H. australis*. *H. perezi* was first described by Chatton and Poisson infecting the portunid crabs *C. maenas* (green crab) and *Liocarcinus depurator* (swimming crab) on the French Mediterranean coast [14]. In Australia, *H. australis* was described in *Portunus pelagicus*, the sand crab (also confusingly known as “blue swimmer crab”) [15]. Crustaceans residing in deep, stenohaline waters, such as *Chionoecetes opilio, C. bairdi, C. tanneri* and *Nephrops norvegicus*, are infected by yet another genotype of *Hematodinium* sp. distinct from *H. perezi* and *H. australis*[16,17]. For many years, data gaps in descriptions of morphology, host range and sequence data led to uncertainty in species designations, [18] resulting in the species of *Hematodinium* infecting blue crabs being referred to generically as *Hematodinium* sp. [12,19,20]. Recent work by Small *et al*. confirms that the *Hematodinium* sp. infecting blue crabs on the US Atlantic coast is *H. perezi*[18,21].

Epizootics of *H. perezi* in blue crab can recur annually and are often localized in coastal bay “hotspots”, characterized by constrained, high salinity, warm waters, suggesting that there are environmental or biotic reservoirs of the parasite [22]. The parasite is found in the hemolymph and tissue of the blue crab where it can multiply rapidly, eventually causing organ failure and crab death [13]. The life cycle of *Hematodinium* sp. is complex and includes trophont and sporont cell types within the host and flagellated dinospores that are occasionally observed within and emerging from infected hosts [23-25]. Recently, Li *et al*. reported on the *in vitro* culture of the entire *H. perezi* life cycle including the dinospore stages [26]. The existence of free-living dinospores suggests that the environment (water or sediment) can be a source of new *H. perezi* infections.

Detection of *Hematodinium* sp. within host tissues can be achieved by histological methods [11,13], or by using vital stains for live parasites in fresh hemolymph [11,14,27]. These methods, however, are impractical for detecting the parasite in environmental samples. Molecular methods, developed for detection of *Hematodinium* spp. in commercially important crustaceans [17,28-31], have the potential to be applied to DNA extracted from environmental samples. Initial research, using either endpoint polymerase chain reaction (PCR) or quantitative PCR (qPCR), targeted the SSU region of the parasite ribosomal RNA gene to assess *Hematodinium* sp. infections in blue crab hemolymph [28,29]. Although these primers were sufficient for detection within blue crab, the sequences targeted were not specific enough for analysis of environmental samples, which may contain DNA of free-living dinoflagellate species that possess SSU gene sequences similar to those of *Hematodinium* sp. [31,32].

There are two frequently encountered challenges when using qPCR to look for organisms in the environment. First, the accuracy of a qPCR assay rests on the ability to produce a reliable standard curve, which is usually based on DNA extracted from a pure culture of the organism itself or on plasmid DNA carrying the target sequence of the organism of interest. Until recently [26], *H. perezi*, cultures were not readily available for use in generating standard curves, and reliable passage of infections from wild isolates posed significant technical hurdles. The second challenge is that, in practice, accuracy of a qPCR method also depends on reproducible extraction of target DNA from environmental samples. An assay based on purified or cloned target DNA may not be representative of how parasite DNA is recovered from environmental samples in which there is a heterogeneous mixture of DNA from other species, in addition to potential qPCR inhibitory compounds. Therefore, to develop methods suitable for environmental assessment and prevalence studies, we sought to design and validate a qPCR assay targeting the species-specific ITS2 region of the rRNA gene, and establish a standard curve using a surrogate for *H. perezi* cells mixed with environmental sediment. We then used this assay to look for *H. perezi* in environmental samples on a temporal and spatial scale.

## Results

### Design of an ITS2-targeted PCR assay for *H. perezi* ex *C. sapidus*

Initial efforts to detect DNA of *H. perezi* in environmental DNA samples using an SSU-targeted qPCR assay [29] indicated that DNA of non-target organisms may produce spurious results. When the Nagle et al. [29] SSU primers (PlaceF, PlaceR, Table 1) were used to amplify DNA extracted from sediment from Parsons Creek, MD (N38.5017˚, W76.2669˚), a low salinity subestuary of the Chesapeake Bay with no record of *H. perezi* infections, a positive qPCR signal was nevertheless obtained (data not shown). The product displayed the expected mobility on agarose gels, but its sequence was an imperfect match to the available sequences for *Hematodinium* sp. such as AF286023 [28], showing a single base substitution relative to *the* GenBank entry. A BLAST database search using the predicted *H. perezi* SSU amplicon returned at least ten non-*Hematodinium* sp. protozoan species that are identical in sequence to the Nagle *et al.*[29] primers and probe, and therefore would be expected to generate positive results. Furthermore, the list of matching organisms (Additional file 1: Table S1) included species found in mid-Atlantic and Gulf coast waters (areas that also harbor *H. perezi*) which demonstrated the need to develop a new primer set.

**Table 1:** Oligonucleotides used in this study

**Oligo-nucleotide**	**Sequence (5′ to 3′)**	**Purpose/target**	**Source**
PlaceF	GGGTAATCTTCTGAAAACGCATCGT	*Hematodinium* SSU	[29]
PlaceR	GTACAAAGGGCAGGGACGTAATC	*Hematodinium* SSU	[29]
D2C	CCTTGGTCCGTGTTTCAAG	Dinoflagellate LSU	[33]
1487 F	CCTGGCTCGATAGAGTTG	*Hematodinium* SSU	[28]
HemITSF2	TCGTAACAAGGTTTCCGTAGG	*Hematodinium* SSU	This study
HemITSR2	GACCCAGCCTTCACGATAAA	*Hematodinium* LSU	This study
M13 F	CGCCAGGGTTTTCCCAGTCACGAC	pGEM-T vector	
M13 R	TCACACAGGAAACAGCTATGAC	pGEM-T vector	
ITS2For	AGGTCTAATGCTTGTTGGCC	*H. perezi* ITS2 qPCR	This study
ITS2Rev	CACTAGTCCGAAAACCTGTG	*H. perezi* ITS2 qPCR	This study
HemITS2 probe	6-FAM-ACCGCTACTCTTCTT-CCGCCCT-BHQ1	*H. perezi* ITS2 qPCR	This study

**Figure 1:**
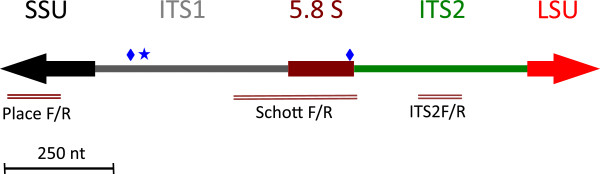
**Schematic representation of *H. perezi *rDNA locus.** Figure shows locations of PCR primer pairs (Table 1), relative to SSU, ITS, 5.8S, and LSU genes as defined in GenBank entry JQ815886. PlaceF/R primers are from Nagle *et al.*[29], and target the SSU gene. Primers Schott F/R are from Pitula *et al*. [34] and amplify the 5.8S gene. Primers ITS2F/R were developed in the current study. The star and diamonds indicate locations of indel and single nucleotide polymorphisms, respectively, between clones of *H. perezi* derived from independent infections of *C. sapidus*. Polymorphisms are annotated in GenBank JQ815886.

Based on the likelihood that amplification of DNA other than *H. perezi* would occur with DNA samples from water or sediment using the Nagle *et al.*[29] primers, we designed a more specific set of primers, targeting the internal transcribed spacer (ITS2) region. To first asses variability in the ITS regions, a 2 kb amplicon of the *H. perezi* ribosomal DNA gene cluster, encompassing the 5′ end of the SSU, entire ITS1-5.8S-ITS2 and partial LSU regions was cloned and sequenced from 4 infected crabs, 2 from Maryland and 2 from Mississippi. Sequences of all clones were very similar, with the exception of a 3 base insertion/deletion (ATA) in the ITS1 region and two single nucleotide polymorphisms in ITS1 and 5.8S regions. A consensus sequence of the region is deposited in GenBank as accession JQ815886, with annotations to identify the locations of inter-crab polymorphisms. A schematic of the locations of individual single nucleotide polymorphisms (SNPs) and the insertion/deletion (indel) is depicted in Figure 1. Sequence polymorphisms did not correlate with geographic origin of the *H. perezi* infections. For example, both forms of the (ATA) indel were found in infections from Maryland and Mississippi. In fact, both variants were found in the DNA extracted from a single infected crab from MD. Direct sequencing of the PCR products obtained from a single infected crab showed mixed bases (double peaks) present at nucleotide positions that showed SNPs or indel between cloned amplicons.

Because we observed SNPs and an indel in the ITS1 and 5.8S regions (even within a single infected crab) but not in the sequences of the ITS2, we targeted the ITS2 region for development of a qPCR assay. Application of a computer algorithm from the CLC Workbench program and visual inspection led to the design of primers HemITS2F and HemITS2R, which produce an amplicon of 99 bp, as well as the HemITS2probe (Table 1) carrying a FAM fluorophore and BHQ quencher (see Methods). When used to amplify a serial dilution of purified plasmid (pES146) carrying the *H. perezi* ITS2 rRNA region, the assay had a typical efficiency of at least 93% and a sensitivity of 13 copies.

### Validation of the qPCR assay using *H. perezi* cell surrogates

**Figure 2:**
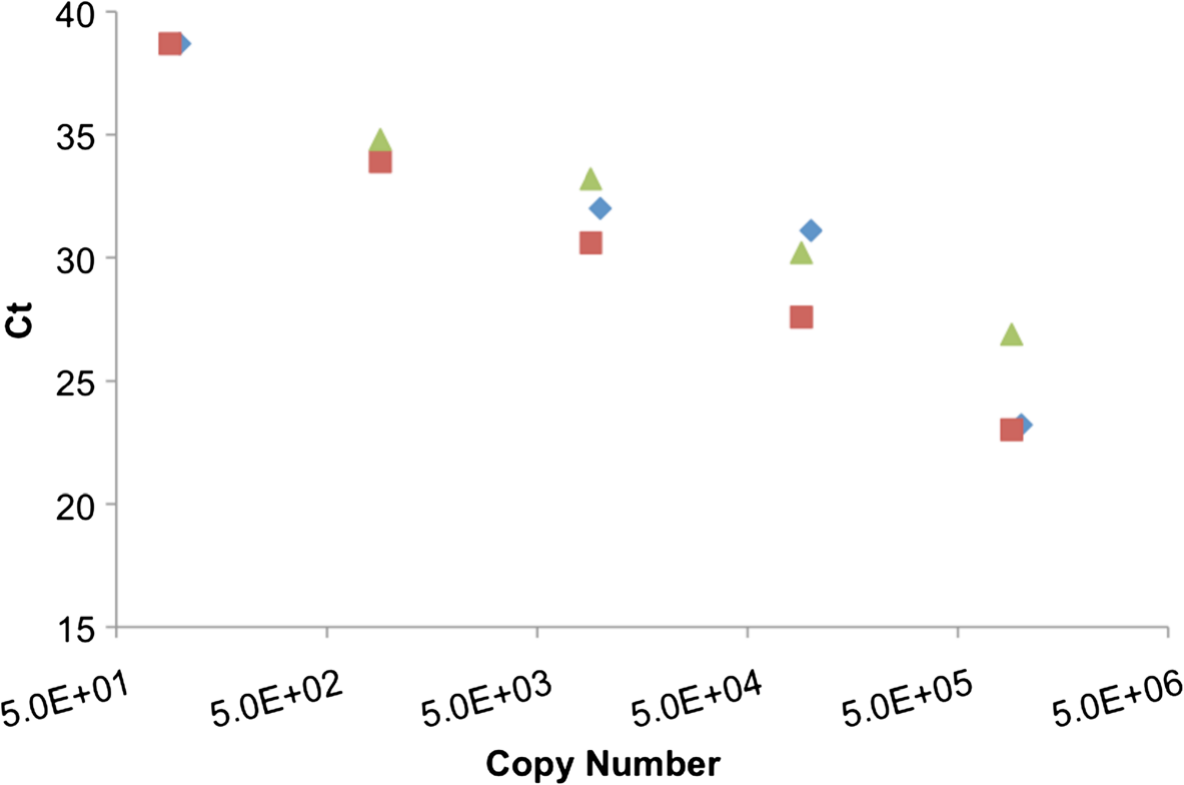
**Comparison of the performance of the *H. perezi *qPCR assay with purified target DNA, and DNA from sediment spiked with a surrogate for the parasite.** Standard curves were generated from 10-fold serial dilutions of DNA corresponding to purified cloned plasmid pES103 DNA (symbol ♦ slope of −3.39, R^2^ = 0.917), DNA prepared with the MoBio kit, from *E. coli* xcontaining plasmid pES103 (symbol ▲, slope of −2.70, R^2^ = 0.979), and DNA prepared from sediment spiked with *E. coli* containing plasmid pES103 (symbol ■, slope of −3.22, R^2^ = 0.993).

To develop a reference applicable to environmental samples, a qPCR standard curve of plasmid DNA was compared to DNA extracted from a dilution series of the *H. perezi* surrogate, and to DNA extracted from sediment spiked with a dilution series of the *H. perezi* surrogate (see Methods). The slopes of each dilution series were −3.39 (purified plasmid), -2.70 (*H. perezi* surrogate), and – 3.22 (sediment with *H. perezi* surrogate) (Figure 2). In order to determine if sediment type and background DNA would have an effect on the efficiency, we also spiked two different types of sediment (sandy and silty) and found that this did not affect the efficiency or sensitivity of the qPCR assay (data not shown). Prior to spiking, sediment samples were shown to be negative for *H. perezi* by ITS2-targeted qPCR.

### Using the qPCR assay to detect *H. perezi* on a spatial scale in sediment in Indian River Inlet, DE

**Figure 3:**
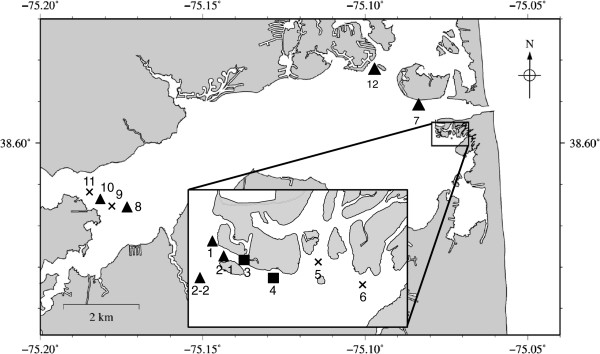
**Map of Indian River Inlet sampling sites.** In 2008, sites sampled were 1, 2–1, 2–2, 3, 4, 5, 7, 12. In 2009, sampling was expanded to all locations (1 through12). Symbols (▲ ■ **X**) refer to 2009 data only. In 2009, sediment sampling was expanded to include all 13 locations, and triplicate samples were taken at each site. Sites from which none of the triplicate samples were positive are indicated by crosses (**X**), sites with one of the triplicate samples positive are indicated with triangles (▲), and sites from which all three triplicates were positive are indicated with squares (■).

On three dates in 2008 (07.01.08, 07.29.08, 12.09.08), eight sites within the Indian River Inlet were sampled (Figure 3). Water and sediment were collected on both July trips; sediment only was collected on the 12.09.08 trip. DNA was extracted from water and sediment, and qPCR assays for *H. perezi* were conducted as described in Methods. Of the 07.01.08 samples, only sediment DNA from site IR7 produced signal by qPCR (Table 2). From the 07.29.08 sampling, three sites produced positive qPCR signal: IR2-1, IR2-2, and IR3. There were no positives among the 12.09.08 sediment samples. None of the DNA extracted from the 0.1 liter water samples from any 2008 site produced ITS2 qPCR signal.

**Table 2:** **Levels of *H. perezi *from Indian River Inlet samples**

**Indian River Environmental samples**				
	**Sampling date**	**Positive sites**	**Sediment type**	**Gene copies/g sediment**
**Sediment**	07.01.08	IR7	sandy clay	1.0E + 04
	07.29.08	IR2-1	silty sand	5.9E + 03
		IR2-2	sand	4.4E + 03
		IR3	silty sand	5.8E + 03
	07.15.09	IR1a	silty sand	2.0E + 02
		IR2-1 c	silty sand	1.5E + 02
		IR3a	silt	1.7E + 03
		IR3b	silt	9.5E + 02
		IR3c	silty sand	2.3E + 02
		IR4a	silty sand	1.7E + 02
		IR4b	silty sand	1.1E + 03
		IR4c	silty sand	3.6E + 02
		IR7a	sand	1.5E + 03
		IR8c	silt	2.2E + 02
		IR10b	silt	2.3E + 02
		IR12b	silt	4.0E + 02
				**Gene copies/L water**
**Water**	07.15.09	IR1	n/a	6.1E + 02
		IR3	n/a	4.0E + 02
		IR11	n/a	1.3E + 02
		IR12	n/a	8.2E + 01

Quantitative PCR was used to assess *H. perezi* in DNA extracted from environmental samples from 13 locations in Indian River Inlet, DE (Figure 3) Samples were collected at indicated dates in July 2008 and 2009. In July 2009, each site was sampled in triplicate, as indicated by suffixes a, b, and c. Results are reported as *H. perezi* ribosomal gene copy number per g sediment and gene copies per L of water. Note that 12.09.08 sediment samples were collected but were all negative.

During the 07.29.08 sampling trip, many dead and dying *C. sapidus* were observed in Indian River Inlet. No mass mortality of other species was detected. Nineteen dead or moribund *C. sapidus*, as well as apparently healthy individuals of two additional crab species (*Ovalipes ocellatus* and *Carcinus maenas*) were collected from the eastern end of Indian River Inlet. Fifteen of the *C. sapidus*, as well as both the *C. maenas* and *O. ocellatus* were found to be positive for *H. perezi* (Table 3)*.* The *C. sapidus* infections displayed from 1.01E + 06 to 1.63E + 08 gene copies per g tissue, while the *C. maenas* and *O. ocellatus* infections were at 1.79E + 06 and 4.79E + 05 gene copies per g tissue, respectively. One of the *C. sapidus* DNA samples showed strong PCR inhibition (failure to amplify even when spiked with 10E + 05 copies of the cloned target).

**Table 3:** **Levels of *H. perezi *in blue crab during an outbreak in Indian River Inlet, DE**

**Collection location**	**Species**	**Gene copies/g tissue**
1	*C. sapidus*	2.53E + 06
1	*C. sapidus*	4.05E + 06
1	*C. sapidus*	4.05E + 06
1	*C. sapidus*	1.04E + 07
1	*C. sapidus*	1.16E + 07
1	*C. sapidus*	4.55E + 07
1	*C. sapidus*	1.43E + 08
1	*C. maenas*	1.79E + 06
2-1	*C. sapidus*	-*
2-1	*C. sapidus*	<10
2-1	*C. sapidus*	<10
2-1	*C. sapidus*	<10
2-1	*C. sapidus*	3.76E + 07
2-1	*C. sapidus*	1.63E + 08
5	*C. sapidus*	1.01E + 06
5	*C. sapidus*	9.88E + 06
5	*C. sapidus*	2.99E + 07
7	*C. sapidus*	3.59E + 07
7	*C. sapidus*	7.54E + 07
7	*C. sapidus*	1.08E + 08
7	*O. ocellatus*	4.79E + 05

* PCR inhibition prevented assessment of this sample.

*H. perezi rRNA gene* copy number per g tissue from crabs collected at Indian River Inlet, DE, on 07.15.08 during an *H. perezi* outbreak.

During the apparent *H. perezi* outbreak, snails (*Ilyanassa obsoleta*) were found to be foraging amongst dead and dying crabs. In addition, grass shrimp (*Palaemonetes pugio*) were abundant in the waters in which the outbreak occurred. DNA of both snails and shrimp (n = 12, each species) were assessed for *H. perezi* using the qPCR assay. Despite their proximity to blue crabs that were infected with *H. perezi*, neither snails nor shrimp were positive by the ITS2 qPCR assay.

Based on the 2008 findings that *H. perezi* DNA was present in sites 2–1 and 3, the 2009 sampling focused on sites 2 and 3, expanding a transect along an east–west line and conducting sampling in triplicate at each location. Additional sample sites were added in the western end of Indian River Inlet, downstream of an electricity generating station (sites IR9-IR12 in Figure 3). In 2009 two sampling trips were conducted, on 06.01.09 and 07.15.09. None of the samples from 06.01.09 were positive for *H. perezi* by the ITS2 assay. In contrast, eight of the 12 sites during the 07.15.09 sampling trip yielded positive signal in at least one of the triplicate samples (Table 2). In six of the sites only one of three replicates was positive (sites 1, 2, 7, 8, 10, 12). However sites IR3 and IR4 produced positive signal from all of the triplicate samples at each site. In 2009, at each location, 1 L of water (instead of 0.1 L as in 2008) was collected for DNA extraction and testing for *H. perezi* using the ITS2 qPCR assay. Four of the sites (IR1, IR3, IR11, IR12) produced signal in this assay (Table 2).

In 2009 no moribund or dead *C. sapidus* were encountered in any of the sampling trips. Collections of *C. sapidus* were neglected in this year; however, two non-crab species were collected opportunistically: the amphipod *Orchestia grillus* (n = 7) and grass shrimp *P. pugio* (n = 10). Neither of these species yielded positive results in the ITS2 *H. perezi* qPCR assay.

### Using qPCR to investigate *H. perezi* incidence on a seasonal scale in Chincoteague Bay

**Figure 4:**
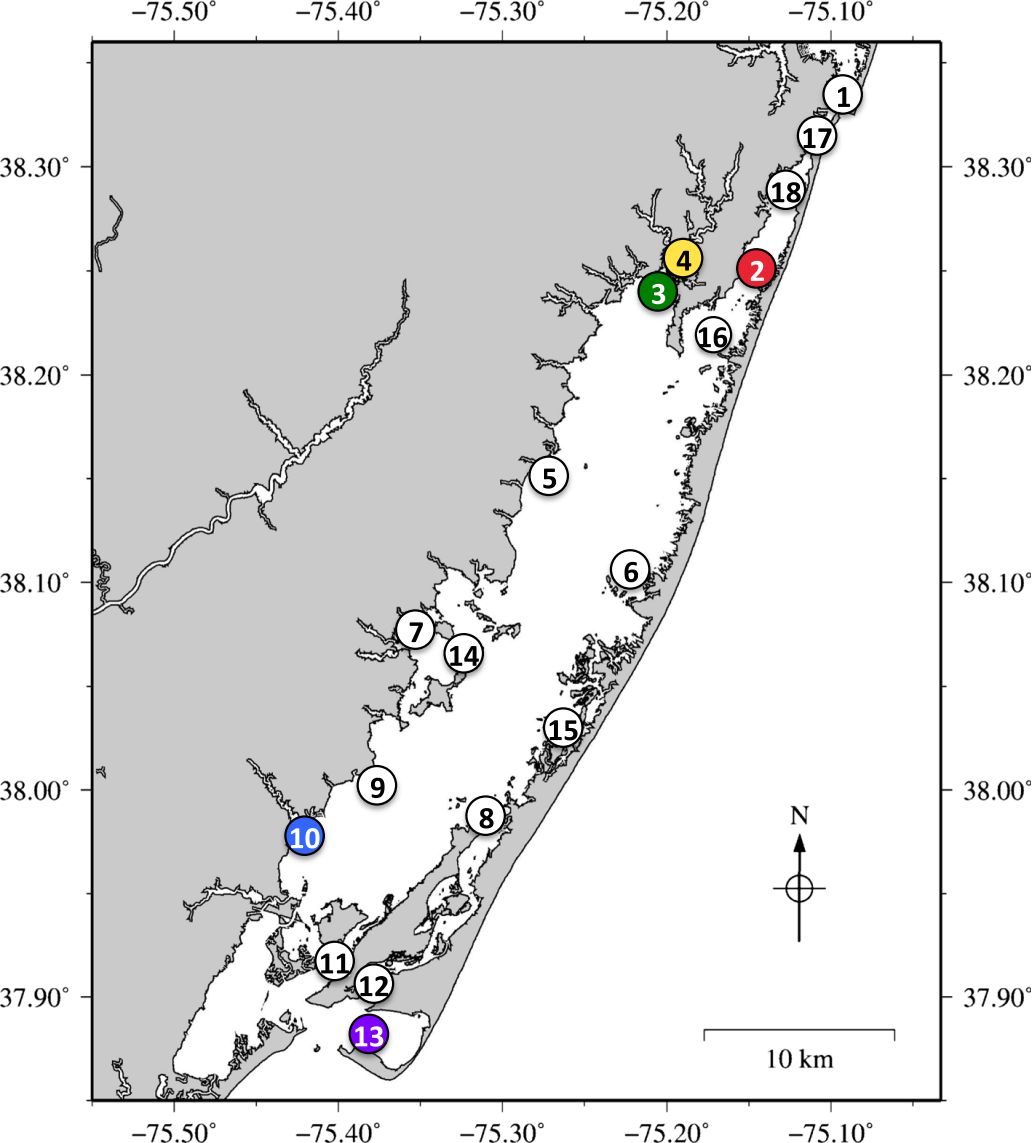
**Map of Chincoteague Bay sampling sites.** From April through November of 2010, sediment and water/plankton samples were collected from 18 sites monitored by the National Park Service water quality program. Sites are represented by numbers in open circles: 1: Commercial Harbor; 2: Verrazano Bridge; 3: Newport Bay; 4: Trappe Creek; 5: Public Landing; 6: Whittington Point; 7: Taylor’s Landing; 8: Wildcat Point; 9: Greenbackville; 10: Sinnickson; 11: Chincoteague Channel; 12: Assateague Channel; 13: Tom’s Cove; 14: Johnson’s Bay; 15: Cedar Island; 16: South Point; 17: Ocean City Inlet; 18: Snug Harbor. Sediment and water/plankton samples in which *H. perezi* DNA was detected more than once are shown as colored circles corresponding to the colors in Figure 5.

**Figure 5:**
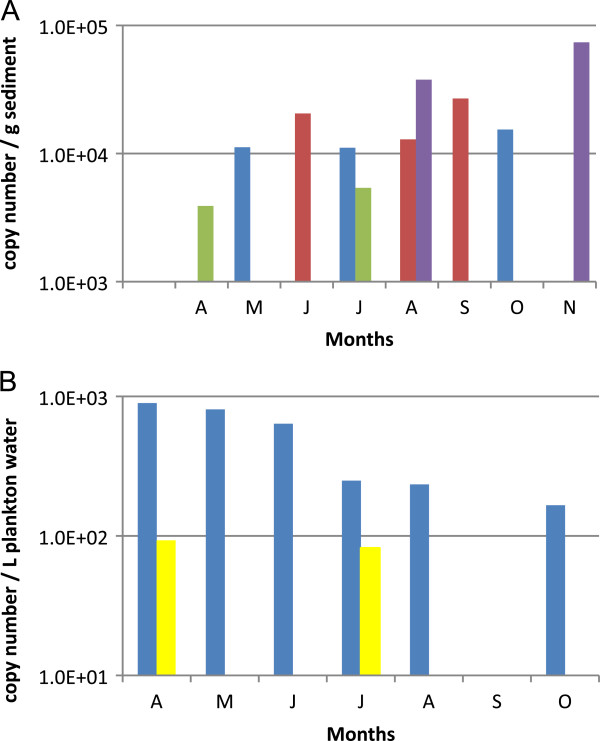
**Temporal variation of the incidence of *H. perezi *DNA in MD and VA coastal bays.** Detection of *H. perezi* in samples from the Chincoteague Bay from April to November 2011. Panel 4**A** shows results from the sediment. Panel 4**B** shows results from water/plankton. Results are displayed as *H. perezi* ribosomal gene copy number per g sediment or per 1 L water/plankton (blue = Sinnickson, green = Verrazano Bridge, red = Newport Bay, purple = Tom’s Cove, yellow = Trappe Creek).

In a study to follow the temporal and large-scale spatial occurrence of *H. perezi* in a coastal bay ecosystem, eighteen sites within Chincoteague Bay were sampled on a monthly basis from April to November of 2010 (Figure 4). Both plankton and sediment were collected at each location, and DNA was extracted for analysis by the ITS2 qPCR assay. Overall, 34 of the 324 Chincoteague Bay water and sediment samples (10.5%) were positive for *H. perezi* DNA by the ITS2 qPCR assay (Additional file 2: Table S2). There was no obvious geographic pattern or concentration of sediment signal in any one region or month. Fifteen of the sites were positive at least once during the nine month study. The three sites that never tested positive for *H. perezi* DNA (Assateague Channel (12), Ocean City Inlet (17), and South Point (16) are in areas with high tidal flux and have mostly sandy sediment. Ocean City and Assateague Channel Inlet are at the constricted north and south ends of the Bay, where water velocity is expected to be highest. Sediment from four sites tested positive for *H. perezi* in two or more months (Figure 5A)*.* These four sites (Verrazano (2), Newport Bay (3), Sinnickson (10), Tom’s Cove (13)), encompass most of the north–south axis of Chincoteague Bay, as well as the east–west axis. The physical characteristics of the four sites are quite diverse: two are near freshwater inputs (Sinnickson and Newport Bay), Tom’s Cove is a shallow bay with no freshwater input, and Verrazano is near deep water in a wide channel between Ocean City inlet and the main Chincoteague Bay. One common feature of all four sites is that the sediment contained silt in addition to clay, loam or sand.

In contrast to the results for sediment, which showed at least one positive sample at most sites, analysis of plankton samples revealed that just eight of the locations were positive by the qPCR assay during the nine month study (Additional file 2: Table S2). Plankton samples from six of these sites tested positive only once, plankton samples from one site tested positive twice (Trappe Creek (4)), and plankton samples from Sinnickson (10), a site on the western side of Chincoteague Bay, were positive for *H. perezi* in six of the nine months sampled in 2010 (Figure 5B).

## Discussion

Along the US Atlantic coast, *H. perezi* infections in blue crabs display an annual peak of prevalence and intensity in late summer or autumn [22]. Conversely, *H. perezi* infections disappear between peaks, and therefore raise the question of whether there is a source (reservoir) of new *H. perezi* infections*.* Possible *H. perezi* reservoirs include other blue crabs, alternate hosts, and the environment. There have been conflicting reports as to the role of cannibalism in transmission [32,35]. *Hematodinium* sp. has been detected in numerous alternate hosts sympatric with (and possible prey for) blue crab [36], but only at low prevalence and intensity [37]. The existence of a water-borne dinospore stage is well documented, and recent work supports a model in which *Hematodinium* spp. may have a resting stage (cyst, or another overwintering stage) that could be a source of re-infection [23,32,34,38].

PCR is a practical technique for investigating the spatial and temporal presence of *H. perezi* in environmental reservoirs. A number of PCR assays have been developed to detect *Hematodinium* spp. DNA, all of which target one of the regions within the ribosomal RNA locus [28,29,31,32,39]. The SSU region of the rDNA locus shares high sequence identity between different species of *Hematodinium* (including the various clades described by others [17,39,40]). The *Hematodinium* sp. SSU also shares significant identity to other, free living, dinoflagellates, as we discovered at the initiation of the present study. As reflected in Additional file 1: Table S1, BLAST analysis revealed that the primers and probe used in Nagle *et al.*[29] are predicted to amplify the SSU sequences from presumptive free-living dinoflagellates on both the Gulf and Atlantic coasts.

More species-specific sequences are found in ITS1 and ITS2 regions, which are transcribed as part of the polycistronic rRNA product, but are not part of the mature ribosomal RNAs (SSU, 5.8S and LSU) [21]. We chose to develop a PCR assay based on the ITS2 region for two reasons: firstly, when rDNA clones derived from several *H. perezi* infections were compared, the ITS2 region displayed few clone-to-clone SNPs and length polymorphisms, as compared to the ITS1 region; and secondly, the intra-species variation of the sequence of the ITS2 region is constrained by its role in processing of the final rRNA molecules. The ITS2 region of the rRNA transcript folds into a cruciform structure that interacts with splicing enzymes. Coleman [41] and others [42] have reported on the advantages of the ITS2 as a molecular feature for taxonomic comparisons between congeneric species. Our finding that a single *H. perezi*-infected crab had two versions of an ITS1 polymorphism could be interpreted either to mean that the crab carried a dual infection of two strains that differ in ITS1 sequence, or that a single strain of *H. perezi* carried both versions, similar to within-strain ITS1 length variation in insects [43].

In this study a surrogate for *H. perezi* was used to assess the efficiency of target DNA recovery from environmental samples. DNA preparations from environmental samples are oftentimes inhibitory to PCR amplification. In place of hypothetical environmental forms, or hard-to-maintain hemolymph stages of *H. perezi,* we used sediment spiked with bacteria carrying the cloned PCR target to validate DNA extractions and PCR assays. Using this approach, we estimated that the PCR method has a detection limit of 13 gene copies per qPCR reaction, which translates to 1300 copies per gram of sediment. However, the minimum number of parasite cells that can be detected by the method is still uncertain, because there is disagreement in the literature as to how many rRNA gene copies there are per *H. perezi* cell. Nagle *et al*. [29], reported up to 10,000 copies/cell, while Li *et al*. [32], reported approximately 300 copies/cell. Interestingly, in a recent study of rRNA gene copy number in a dinoflagellate, Perini *et al.*[44] found that, depending on strain and stage in culture, *Ostreopsis cf. ovata* may possess from 81 to 8488 copies/cell of the LSU gene. This example provides one possible explanation for why the two estimates of *H. perezi* rRNA gene copy number may be so divergent. It would be worthwhile to assess the number of rRNA genes per *H. perezi* cell from a number of different isolates of the parasite and different developmental stages within the host. The recent development of *in vitro* culture for *H. perezi* by Li *et al*. [26] should provide valuable material for an assessment of rRNA gene copy number through different life stages.

Although the question of how many copies of the rRNA locus are present per cell of *H. perezi* was not addressed in the current study, the field data we obtained is pertinent to the issue. Many of the environmental samples in the current study contained from several hundred to several thousand ITS2 targets per gram sediment or per liter water/plankton. These values are more consistent with the gene copy numbers of Li *et al*. [32] unless we postulate that DNA recovery was poor, or that naked *H. perezi* DNA (representing less than one cell) was present in environmental samples. Using dilutions of ethanol-preserved *H. perezi* trophonts, we have also generated qPCR data that are more consistent with the lower number of gene copies/cell (Hanif and Schott, unpublished). With the lower gene copy/cell in mind, it should be recognized that samples that were negative by PCR (including replicate samples at sites that also had at least one positive) may indeed carry *H. perezi* DNA below the limit of detection. At 300 copies per cell, however, our detection limit of 1300 copies per gram sediment would translate to 4 cells. Collections of 0.25 gram would therefore be at the theoretical limit of one cell per sample. Sampling within sediment that had such a low density of *H. perezi* cells would therefore be expected to produce many samples that fail to capture a cell.

There are several themes that emerge from the application of the *H. perezi* qPCR assay to the coastal bay study. 1) *H. perezi* signal was widely detected throughout both coastal bays, and a potential sediment hotspot was detected in Indian River Inlet. 2) *H. perezi* DNA was detected in water/plankton samples from geographically dispersed areas in both coastal bays and was temporally consistent in prevalence at some locations such as Sinnickson in Chincoteague Bay, where it was detected during all seasons in which samples were collected. 3) *H. perezi* DNA was not found in non-portunid invertebrates, even those collected in the vicinity of a mass mortality of blue crabs infected with *H. perezi*.

Repeated detection of *H. perezi* DNA in sediment is consistent with the hypothesis that there is a sediment reservoir for the parasite. Hamilton *et al.*[45] suggested that, like other dinoflagellates, *Hematodinium* sp. may have a resting stage outside of its host as a part of its life cycle. Though a cyst or resting stage has not been observed by microscopy, molecular methods have been used to detect parasite DNA in sediment samples [21,29]. Frischer *et al*. [23] presented evidence that the dinospore stage could establish infections in naïve blue crabs upon *in situ* exposure to water and sediment. Li *et al.*[46] showed that *H. perezi* amoeboid trophonts begin to lose viability after 24 h in seawater, but that dinospores survive up to seven days in aquaria [32]. *H. perezi* dinospores may be a precursor to a resting stage, which could accumulate in undisturbed sediments. Survival time of dinospores and hypothetical resting stages may vary according to factors such as predation, destruction by bacteria or environmental conditions [47,48].

Plankton DNA samples from Sinnickson were positive for *H. perezi* in six of the nine months sampled (Additional file 2: Table S2 and Figure 5). Finding evidence of the parasite in the water/plankton, which is highly mobile, implies the existence of a persistent environmental source. Sediment could be that source, consistent with the finding of *H. perezi* DNA in sediment from Sinnickson in three of the nine dates sampled, and in all seasons. The other site with multiple positive plankton samples (two months out of nine months sampled) was Trappe Creek, which, like Sinnickson, has a sizeable freshwater input. Trappe Creek is close to, and upstream of, Newport Bay, which had positive sediment three of the nine months sampled. A possible mode of *H. perezi* DNA accumulation in sediments and water/plankton of some areas and not others is through tidal cycles that cause water (and plankton) to oscillate in/out of the small rivers feeding coastal bays. This is in contrast to the channel areas that have faster currents and have less slack water than may be found in the broad mouths of rivers. This is reflected in the sediment types in each area: only sand is present in the channels while silt-containing sediments are adjacent to broad river outlets.

The presence of *H. perezi* DNA in 2009 water and sediment samples obtained from Indian River Inlet was similar to observations (above) in Chincoteague Bay in 2010. Most of the Indian River Inlet *H. perezi* signal (including the hotspot) was present in silty sediment with high organic matter content, located in a backwater that experienced relatively little tidal flushing. On 07.29.08, *H. perezi* was not detected in water from Indian River Inlet, although sampling coincided with what appeared to be a *H. perezi* outbreak in blue crabs. However, the volume of water collected for analysis at that date (100 ml) was probably insufficient for detection of low amounts of parasite DNA. In 2009, when the volume of water sampled was increased to 1 L, qPCR analysis yielded 82 to 613 gene copies per liter. If the 2008 samples had contained parasite DNA at this abundance, a 100 ml water sample would have had been below the limit of detection for the qPCR assay.

On 07.29.08, the presence of many of dead and dying crabs that contained *H. perezi* gave us an opportunity to examine the water as well as other aquatic species during a parasite outbreak. *H. perezi* was detected in the muscle tissue of both lady crab (*O. ocellatus*) and green crab (*C. maenas*), which is in agreement with previous reports of *Hematodinium* sp. in these two species [39,40]. Both of these crabs were collected (and appeared healthy) during the outbreak, and could represent incidental carriers or reservoir hosts. None of the grass shrimp, amphipods, or snails (grazing on shells of dead *C. sapidus*) were positive for *H. perezi* DNA, which argues that these are not major *H. perezi* reservoirs. This is consistent with the study by Pagenkopp *et al.*[37], who found no *H. perezi* in over 200 *Palaemonetes* sp. shrimp, and only 3% prevalence in caprellid amphipods. It was concluded that these were not likely sources of blue crab infections of *H. perezi.* Pagenkopp *et al.*[37] did however suggest that spider crabs (*Libinia dubai*), which we did not collect in our study, could serve as a biotic reservoir based on a prevalence of 17%.

Lastly, another possibility is that blue crabs with pre-patent *H. perezi* infections could act as reservoirs of the parasite. Blue crabs collected from winter trawls in Virginia, USA have been reported to be infected with *H. perezi*[22,37]. Given that blue crabs are cannibalistic and feed heavily after hibernation, it has been suggested that infections could be transmitted via this mode. However, there have been conflicting results with cannibalism studies in laboratory settings. When Walker et al. [35] fed blue crabs fed infected conspecific tissue, they successfully transmitted the parasite to 7 of 11 blue crabs. A more recent study by Li *et al.*[46] showed a lack of transmission to naïve blue crabs and amphipods via cannibalism. More research is needed to elucidate if transmission may occur by entry of dinospores through carapace, gills, or breaks in integument, either while scavenging on infected prey or from environmental sources such as the water column or sediment.

## Conclusions

Application of a species-specific *H. perezi* qPCR assay to DNA extracted from coastal bay sediment, plankton, and water samples suggests that *H. perezi* is continuously present in Delmarva coastal bays, and may present a source of transmission. These assays show that *H. perezi* DNA in sediments and water in these coastal bays is both temporally and spatially variable. The accumulation of parasite DNA in sediments or “hotspots” may be a consequence of parasite cells settling out of the water column, such as in retentive areas with silty sediments. There are gaps in our understanding of the natural history of the parasite as well as the mode of environmental or biological transmission. Filling some of those information gaps can be accomplished by using the qPCR methodology in a more focused study of the presence of parasite DNA in the environment, paired with detailed studies of water and sediment movements in the vicinity of one or more hotspots. A better understanding of the parasite’s life cycle and modes of transmission will help managers provide more effective fishery forecasts based on environmental conditions.

## Methods

### Sources of *Hematodinium* sp. infected blue crabs and DNA extractions

*Hematodinium* sp.-infected blue crabs and ethanol-fixed blue crab hemolymph from Mississippi were kindly provided by colleagues at the Gulf Coast Research Lab (GCRL, Ocean Springs, MS; Noah Zimmerman, Jeff Lotz) and shipped to the Institute of Marine and Environmental Technology (IMET) by overnight courier. Blue crabs infected with *Hematodinium* sp., obtained from the MD coastal bays, were transported to IMET in coolers and frozen after arrival. Originally identified by the appearance of parasites in fresh hemolymph, all *Hematodinium* sp. infections were verified by PCR amplification using the primers described in Nagle *et al.*[29] or Gruebl *et al*. [28].

DNA was extracted from hemolymph of confirmed *Hematodinium* sp.-infected crabs using the Qiagen DNAEasy Blood and Tissue Kit (Qiagen, Valencia, CA, Cat. # 69506). For DNA extractions from field-collected samples of crabs and other invertebrates, the MoBio Tissue Kit was employed (MoBio, Carlsbad, CA, cat. # 2334). DNA was extracted from water and sediment samples using the MoBio Ultraclean Soil kit (cat. #12800), which includes mechanical tissue disruption. DNA was quantified by spectrophotometry (Nanodrop Inc., Wilmington, DE).

### Amplification and cloning the ITS1-5.8S-ITS2 region of the ribosomal gene cluster

Using Taq polymerase (ExTaq, Takara Bio, Inc., Otsu, Shiga, Japan), and primers 1487 F and D2C (Table 1), DNA fragments encompassing the ITS1-5.8S-ITS2 region were amplified from genomic DNA preparations of a Mississippi and Maryland *H. perezi*- infected blue crab. *Hematodinium*-specific primer 1487 F [28] and a generic eukaryotic primer D2C [33] were used to amplify and clone a ~2 kb region of the rRNA gene cluster from DNA extracted from 4 individual *H. perezi*-infected crabs from both Maryland and Mississippi. Thermocycling conditions were 95°C 5 min, followed by 30 cycles of 94°C, 25 sec; 54°C, 30 sec; 72°C, 3 min. The resulting ~2 kb fragment was ligated to pGEM-T according to manufacturer’s instructions (Promega Corp., Madison, WI) and transformed into *E. coli* strain JM109 (Promega Corp.). Sequencing was performed on a minimum of 3 clones from each infected crab (Gulf or Atlantic), using a combination of primers originating in the pGEM-T vector sequence and cloned *H. perezi* DNA (see Table 1: primers 1487 F, M13F, M13R, HemITSF2, and HemITSR2). Sequencing was conducted using Big Dye Terminator reagents (Applied Biosystems, Inc., Carlsbad, CA) and analyzed with the ABI 3130 XL Genetic Analyzer (Applied Biosystems, Inc.). Sequences were aligned using Sequencher (Genecodes Corp, Ann Arbor, MI) and CLC DNA Workbench (CLC Bio, Aarhus, Denmark). From assembled consensus sequences, additional primers were designed to amplify specific segments of the ITS1-5.8S-ITS2 region to provide additional sequence depth [34]. A consensus sequence of 2056 nucleotides was derived from the assembly and has been submitted to GenBank as accession JQ815886.

### Development of ITS2-targeted qPCR assay

BLAST analyses of the consensus sequence were used to identify boundaries between SSU, ITS1, 5.8S, ITS2 and LSU regions, as compared to cognate sequences in GenBank [49]. Based on the ~280 bp ITS2 region, PCR primers ITS2For and ITS2Rev (Table 1) were designed (CLC bioinformatics software). A TaqMan probe (HemITS2probe) was designed, carrying 6-FAM and BHQ1 as reporter and quencher dyes, respectively. Using the above primers at 400 nm, probe at 300 nm, Taq-pro complete (Denville Scientific, Metuchen, NJ) with 2.5 mM final MgCl_2_ and 0.25 mg/ml BSA (Idaho Technologies, Salt Lake City, Utah), thermocycling was carried using the following conditions: initial heating to 95°C, 5 min; followed by 95°C, 15 sec; 58°C, 30 sec, 45 cycles. For routine analyses, thermocycling was performed on 1 μl of DNA (10 to 50 ng) using an Applied Biosystems Fast7500 thermocycler (Indian River Inlet samples) or Bio-Rad iCycler iQ Optical Model (Chincoteague Bay samples). Cross-checking with a dilution series of the same preparation of plasmid-borne target DNA confirmed that the assay performed with the same sensitivity on both iCycler and Fast7500 thermocyclers.

### Sensitivity and Specificity of the ITS2 assay

The sensitivity of the ITS2 assay was investigated by conducting qPCR on a serial dilution of plasmid pES103, which carries the 2 kb amplicon produced from primers 1487F and D2C, encompassing the 3’ end of the SSU gene as well as complete ITS1, 5.8S, ITS2 and partial LSU genes. An amplicon of the ITS2 region, produced by PCR amplification of pES103 using primers HemITS2for and HemITS2rev, was produced and cloned in pGEM-T to create plasmid pES146. Based on spectrophotometric measurements of plasmid concentration, a series of samples from 13 to 1.3 × 10E + 06 copies were prepared in nuclease free water. Quantitative PCR was conducted on 1 μl of each dilution in triplicate and the results plotted as Ct versus copy number. Slope and R^2^ of the standard curve were calculated using the ABI software (Applied Biosystems 7500 Fast Real-Time PCR system).

### Validation of the qPCR assay using *H. perezi* cell surrogates

Because *H. perezi* cells were not easily obtainable, it was necessary to develop an *H. perezi* surrogate to use in the validation of the assay. This cell surrogate consisted of *E. coli* strain JM109 carrying the plasmid pES103 (ITS1-5.8S-ITS2 regions). The *H. perezi* surrogate was grown in LB + AMP liquid broth medium overnight. Based on OD_A600_ of 1.9 = 1 × 10^9^ cells ml^-1^ we made a 10 fold serial dilution of the overnight culture that was calculated to have from 3 to 3 × 10^5^ bacteria/microliter to spike into sediment. The bacteria cell number was also checked by plating overnight culture on LB + AMP plates. DNA extraction of the *H. perezi* surrogate was done using the MoBio Tissue and Cells DNA extraction kit per manufacturer’s instructions. For spiked sediment, the serially diluted *H. perezi* surrogate was added to sediment from Baltimore Harbor (and shown to be negative for *H. perezi* by ITS2 qPCR). DNA was then extracted from each spiked sediment sample using the MoBio Soil DNA Extraction kit per manufacturer’s instructions. We used the qPCR assay to compare slopes and limits of detection using 1 μl of DNA.

It has been reported that using linearized target DNA may amplify more than one cycle earlier than circular cDNA for a given amount of input DNA, resulting in a miscalculation of gene copy number in unknown samples [50]. To address this issue we compared the slopes and limits of detection of plasmid pES146 in the linear and circular conformations. We found that the linearized target and intact plasmid gave rise to similar Ct numbers compared to those from use of circular DNA (data not shown).

### Environmental sample collection and DNA extractions

#### Sampling locations and seasons

Sampling was conducted at 13 sites in the Indian River Inlet during 2008 and 2009 (Table 2 and Figure 3). In 2008 (07.01.08, 07.29.08, 12.09.08) single samples were collected, while in 2009 (07.15.09), Indian River Inlet sediment samples were taken in triplicate. At all dates, single water samples were taken. Sampling was conducted at 18 sites in and near Chincoteague Bay (Figure 4) between April and November of 2010, in conjunction with the National Park Service water quality monitoring program. The only exception was September, when only three sites were sampled: Verrazano Bridge (Site 2), Newport Bay (Site 3), and Public Landing (Site 5). Maps of sampling were generated with The Generic Mapping Tools v. 4.5.9 [51] using hydrography data retrieved with National Map 2.0 Viewer (U.S. Geological Survey; http://viewer.nationalmap.gov).

#### Biotic samples

Estuarine invertebrates were collected 07.29.08 in Indian River Inlet near sites 1, 2, 5, and 7 at low tide by hand or using long handled nets. In the field, individual animals were placed in labeled ziplock bags and kept on ice until transport to the lab, where they were stored at −20°C. DNA was extracted using the MoBio Cell and Tissue Kit. From snails (*I. obsoleta*), DNA was extracted from a cross section of the viscera that included the stomach or intestine. From grass shrimp (*P. pugio*), DNA was extracted from a cross section of the abdomen that included the intestine. From crab species (*C. sapidus*, *C. maenas*, *O. ocellatus*), DNA was extracted from walking leg or back fin muscle tissue.

#### Sediment

The top 3–5 cm of sediment was collected within Indian River Inlet, DE and Chincoteague Bay, MD and VA. In shallow water (DE), sediment was collected directly with a 50 ml conical tube. In water over 1.5 m deep, a petit ponar grab was used to obtain sediment, and the top layer collected with a 50 ml conical tube. All samples were maintained on ice for transport to the laboratory. Sediment samples were mixed with an equal volume of 15 ppt sterile artificial seawater (Crystal Sea, Marinemix, Baltimore, MD) and 0.5 ml aliquots were frozen at −20°C for up to 6 months until DNA extractions were performed.

DNA was extracted from Indian River Inlet sediment using the MoBio UltraClean Soil DNA kit following manufacturer’s recommendations, with the exception that DNA was eluted from the purification matrix in two aliquots of nuclease free water rather than a single elution. DNA preparations were divided into two aliquots and stored at −80°C. DNA was extracted from MD and VA coastal bays sediment using the Sureprep (Fisher Bioreagents) soil DNA isolation kit.

#### Water

In Indian River Inlet, surface water samples were collected using 1, 2, and 4 L bottles. Water was maintained at 0–4°C until further processing. For 2008 samples, 60 ml aliquots were filtered onto 2.5 cm diameter 1 μM filters (Millipore) using a Swinnex cartridge. DNA was extracted from filters using the MoBio UltraClean Soil DNA kit. For 2009 samples, water was centrifuged at 500 × g for 30 min to sediment particles, and the entire pellet was extracted with the MoBio UltraClean Soil DNA as described above. In Chincoteague Bay, water was sampled using a 30 cm diameter plankton net, towed at ~3 knots for 3 min, resulting in an effective sampling volume of ca. 25 m^3^. The collected material was saved in 500 ml seawater, refrigerated, and transported to the laboratory where 200 μl was used for DNA extractions using the Illustra tissue and cells GenomicPrep™ DNA kit (GE Healthcare, Piscataway, NJ).

## Competing interests

The authors declare that they have no competing interests.

## Authors’ contributions

AH conducted 2008 and 2009 DE field research, conducted PCR assays, and participated in data interpretation and drafting the manuscript. ES conducted project management, data analyses, and drafted the manuscript. JP and WD conducted 2010 MD and VA coastal bay field research. RJ contributed to project planning and management, and manuscript preparation. HB designed and validated the ITS2-targeted primers and assessed field samples. GM contributed *H. perezi*-infected blue crab samples and participated in project planning. ES, AH, HB, JP, GM and RJ contributed to the manuscript. All authors read and approved the final manuscript.

## Supplementary Material

Additional file 1: Table S1 Selected BLAST hits with 100% identity to SSU-targeted primers and probe used in Nagle et al. (2009). BLAST analysis was conducted with the region of *H. perezi* DNA (GenBank accession JQ815886) targeted by Nagle et al. primers and probe (Figure 1). The GenBank accession number for sequences that showed perfect matches to forward and reverse primers and probe sequences are listed. Organism and location descriptions are taken from GenBank entries for each sequence. Two of the accessions (JF791095, FJ914413) are from Atlantic waters in which *H. perezi* also occurs. Click here for file

Additional file 2: Table S2 List of VA coastal bay sediment and water/plankton samples positive for *H. perezi* DNA 2010. Listed are sites at which *H. perezi* was detected in the environment (either water/plankton or sediment) over the course of the survey. These positive identifications represent 34 of the 324 samples taken throughout the sampling period. Triplicate samples of sediment were collected at each site, indicated by the letters a, b, and c. Click here for file
