# Supplementary material for: Variation in spatial and temporal incidence of the crustacean pathogen Hematodinium perezi in environmental samples from Atlantic Coastal Bays
Source: Aquat Biosyst. 2013 May 4;9:11. doi: 10.1186/2046-9063-9-11 (PMC3651331; doi:10.1186/2046-9063-9-11)
Supplement: Additional file 1: Table S1 — Selected BLAST hits with 100% identity to SSU-targeted primers and probe used in Nagle et al. (2009). BLAST analysis was conducted with the region of H. perezi DNA (GenBank accession JQ815886) targeted by Nagle et al. primers and probe (Figure 1). The GenBank accession number for sequences that showed perfect matches to forward and reverse primers and probe sequences are listed. Organism and location descriptions are taken from GenBank entries for each sequence. Two of the accessions (JF791095, FJ914413) are from Atlantic waters in which H. perezi also occurs. [file 2046-9063-9-11-S1.doc]

**Additional file 1: Table S1.** **Selected BLAST hits with 100% identity to SSU-targeted primers and probe used in Nagle et al. (2009).** BLAST analysis was conducted with the region of *H. perezi* DNA (GenBank accession JQ815886) targeted by Nagle et al. primers and probe (**Figure 1**). The GenBank accession number of sequences that showed perfect matches to forward and reverse primers and probe sequences are listed. Organism and location descriptions are taken from GenBank entries for each sequence. Two of the accessions (JF791095, FJ914413) are from Atlantic waters in which *H. perezi* also occurs.

| **GB number** | **Species** | **Habitat** |
| --- | --- | --- |
| HQ438156 | Uncultured marine alveolate clone North_Pole_ SW70_28 | North pole |
| JF791095 | *Leucocryptos marina* | Hypoxic Gulf of Mexico |
| JF275463 | Uncultured dinoflagellate clone plate95f11.g1 | Columbia River estuary |
| GU825000 | Uncultured eukaryote clone AA3F14RJ1B04 | Cariaco Basin, Caribbean |
| FJ914413 | Uncultured marine dinoflagellate clone B11 | Southeastern North Carolina |
| GU825563 | Uncultured eukaryote clone BCI5F14RJ3H06 | Cariaco Basin, Caribbean |
