# Supplementary material for: Variation in spatial and temporal incidence of the crustacean pathogen Hematodinium perezi in environmental samples from Atlantic Coastal Bays
Source: Aquat Biosyst. 2013 May 4;9:11. doi: 10.1186/2046-9063-9-11 (PMC3651331; doi:10.1186/2046-9063-9-11)
Supplement: Additional file 2: Table S2 — List of VA coastal bay sediment and water/plankton samples positive for H. perezi DNA 2010. Listed are sites at which H. perezi was detected in the environment (either water/plankton or sediment) over the course of the survey. These positive identifications represent 34 of the 324 samples taken throughout the sampling period. Triplicate samples of sediment were collected at each site, indicated by the letters a, b, and c. [file 2046-9063-9-11-S2.doc]

**Additional file 2: Table S2. List of VA coastal bay sediment and water/plankton samples positive for *H. perezi* DNA 2010**. Listed are sites at which *H. perezi* was detected in the environment (either water/plankton or sediment) over the course of the survey. These positive identifications represent 34 of the 324 samples taken throughout the sampling period. Three samples of sediment were collected at each site. The letters a, b, and c represent which replicate sample from a specific site tested positive.

| **Maryland Coastal Bays Environmental Samples** | | | | |
| --- | --- | --- | --- | --- |
| **Sample type** | **Sampling month** | **Positive Sites** | **Sediment Type** | **Gene copies/g sediment** |
| **Sediment** | April | Verrazano Bridge (a) | sandy silt | 3.9E+3 |
|  |  | Snug Harbor (b) | sandy | 1.3E+4 |
|  |  |  |  |  |
|  | May | Sinnickson (a) | sandy silt | 7.1E+3 |
|  |  | Sinnickson (b) | sandy silt | 9.8E+3 |
|  |  | Sinnickson (c) | sandy silt | 1.7E+4 |
|  |  |  |  |  |
|  | June | Newport Bay (a) | silt loam | 1.4E+4 |
|  |  | Newport Bay (b) | silt loam | 2.7E+3 |
|  |  |  |  |  |
|  | July | Verrazano Bridge (b) | sandy silt | 5.4E+3 |
|  |  | Sinnickson (a) | sandy silt | 1.5E+4 |
|  |  | Sinnickson (b) | sandy silt | 8.7E+3 |
|  |  | Sinnickson (c) | sandy silt | 9.4E+3 |
|  |  |  |  |  |
|  | August | Whittington Point (a) | sandy clay | 1.3E+4 |
|  |  | Newport Bay (a) | silt loam | 1.3E+4 |
|  |  | Tom’s Cove (a) | silt clay | 5.4E+4 |
|  |  | Tom’s Cove (b) | silt clay | 2.2E+4 |
|  |  | Trappe Creek (a) | silt loam | 2.1E+4 |
|  |  |  |  |  |
|  | September | Newport Bay (a) | silt loam | 3.7E+4 |
|  |  | Newport Bay (b) | silt loam | 1.7E+4 |
|  |  |  |  |  |
|  | October | Sinnickson (a) | sandy silt | 1.5E+4 |
|  |  |  |  |  |
|  | November | Tom’s Cove (b) | silt clay | 7.4E+4 |
|  |  |  |  |  |
|  |  |  |  | **Gene copies/L water** |
| **Water/ Plankton** | 04.10 | Trappe Creek |  | 9.3E+1 |
|  |  | Sinnickson |  | 9.0E+2 |
|  |  | Johnson’s Bay |  | 6.4E+1 |
|  |  |  |  |  |
|  | 05.10 | Sinnickson |  | 8.1E+2 |
|  |  |  |  |  |
|  | 06.10 | Newport Bay |  | 2.7E+2 |
|  |  | Sinnickson |  | 6.4E+2 |
|  |  | Taylor’s Landing |  | 3.3E+2 |
|  |  |  |  |  |
|  | 07.10 | Sinnickson |  | 2.5E+2 |
|  |  | Trappe creek |  | 8.2E+1 |
|  |  | Ocean City Inlet |  | 6.9E+1 |
|  |  | Cedar Island |  | 62.E+2 |
|  |  |  |  |  |
|  | 08.10 | Sinnickson |  | 2.3E+2 |
|  |  |  |  |  |
|  | 09.10 | Public Landing |  | 5.3E+2 |
|  |  |  |  |  |
|  | 10.10 | Sinnickson |  | 1.7E+2 |
